# Supplementary material for: Detection of new pioneer transcription factors as cell-type-specific nucleosome binders
Source: eLife. 2024 Jan 31;12:RP88936. doi: 10.7554/eLife.88936 (PMC10945518; doi:10.7554/eLife.88936)
Supplement: Supplementary file 1. [file elife-88936-supp1.docx]

**Supplementary file 1—table 1.** Top 25% transcription factors found by the enrichment analysis by calculating the binding motif enrichment on nucleosomal regions (NRs) compared to nucleosome-depleted regions (NDRs).

| TF name | Cell line | | Enrichment score | TF name | Cell line | Enrichment score |
| --- | --- | --- | --- | --- | --- | --- |
| ATF2 | HepG2, K562 | 0.65, 0.43 | | **MNT** | HepG2 | 0.25 |
| ATF3 | K562 | 0.22 | | **NEUROD1** | MCF-7 | 0.15 |
| ATF4 | K562 | 0.46 | | **NFATC3** | K562 | 0.14 |
| ATF7 | HepG2, MCF-7 | 0.59, 0.37 | | **NFE2** | K562 | 0.18 |
| BCL6 | K562 | 0.16 | | **NFE2L1** | K562 | 0.13 |
| CEBPB | K562, HepG2, HeLa-S3, MCF-7, H1 | 0.47, 0.26, 0.25, 0.24, 0.19 | | **NFE2L2** | HepG2, HeLa-S3 | 0.21, 0.13 |
| CEBPG | K562, MCF-7 | 0.52, 0.37 | | **NFIB** | MCF-7 | 0.22 |
| CREB1 | HepG2, MCF-7 | 0.26, 0.25 | | **NFIC** | K562 | 0.13 |
| CTCF | HeLa-S3 | 0.13 | | **NFIX** | K562 | 0.31 |
| CUX1 | MCF-7, K562 | 0.38, 0.23 | | **NFYB** | HeLa-S3 | 0.14 |
| ESR1 | MCF-7 | 0.19 | | **NR2C2** | HeLa-S3 | 0.15 |
| ESRRB | K562 | 0.14 | | **NR2F2** | MCF-7 | 0.15 |
| FOS | MCF-7, HeLa-S3 | 0.20, 0.15 | | **ONECUT1** | HepG2 | 0.19 |
| FOSL1 | K562 | 0.14 | | **PBX2** | K562 | 0.27 |
| FOXA1 | MCF-7, K562 | 0.21, 0.16 | | **PKNOX1** | K562, MCF-7 | 0.13, 0.13 |
| FOXA2 | HepG2 | 0.15 | | **RBPJ** | K562 | 0.16 |
| FOXA3 | K562 | 0.20 | | **REST** | HeLa-S3, H1 | 0.26, 0.14 |
| FOXO4 | K562 | 0.19 | | **RFX1** | MCF-7, K562 | 0.20, 0.18 |
| GATA3 | MCF-7 | 0.20 | | **RFX5** | HeLa-S3 | 0.18 |
| HLF | HepG2 | 0.31 | | **SPI1** | K562 | 0.14 |
| HMBOX1 | K562 | 0.24 | | **SREBF1** | MCF-7 | 0.23 |
| IKZF1 | HepG2 | 0.97 | | **SREBF2** | HeLa-S3 | 0.15 |
| IRF1 | K562 | 0.19 | | **SRF** | H1, K562, MCF-7, HepG2 | 0.30, 0.20, 0.15, 0.14 |
| IRF2 | K562 | 0.15 | | **STAT3** | HeLa-S3 | 0.14 |
| IRF3 | HeLa-S3 | 0.22 | | **TCF7L2** | HeLa-S3, MCF-7 | 0.19, 0.17 |
| IRF9 | K562 | 0.18 | | **TFE3** | K562 | 0.26 |
| ISX | HepG2 | 0.14 | | **USF1** | H1, HepG2 | 0.18, 0.15 |
| JUN | HepG2, HeLa-S3 | 0.18, 0.16 | | **USF2** | HeLa-S3 | 0.19 |
| JUND | HepG2, HeLa-S3 | 0.17, 0.15 | | **ZBTB33** | MCF-7 | 0.14 |
| LEF1 | K562 | 0.13 | | **ZKSCAN1** | HeLa-S3, HepG2, K562, MCF-7 | 0.36, 0.25, 0.25, 0.20 |
| MAFF | HepG2, K562, HeLa-S3 | 0.49, 0.41, 0.35 | | **ZNF24** | K562, HepG2 | 0.26, 0.15 |
| MAFG | K562, HepG2 | 0.36, 0.14 | | **ZNF274** | HeLa-S3, K562, HepG2 | 5.40,1.35,0.14 |
| MAFK | HepG2, HeLa-S3, MCF-7, H1, K562 | 0.37, 0.25, 0.24, 0.16, 0.15 | | **ZNF282** | HepG2 | 0.16 |
| MEF2A | K562 | 0.15 | | **ZNF382** | HepG2 | 0.30 |
| MEF2D | K562 | 0.18 | | **ZNF460** | HepG2 | 0.13 |
| MITF | K562 | 0.24 | | **ZNF652** | HepG2 | 0.14 |

**Supplementary file 1—table 2.** Predicted potential pioneer factors from enrichment analysis using all identified NRs and NDRs in open chromatin regions. Top 25% transcription factors found by the enrichment analysis by calculating the binding motif enrichment on nucleosomal regions (NRs) compared to nucleosome-depleted regions (NDRs) and are significantly expressed (RPKM >=10) in corresponding cell lines.

| TF name | Cell line | Enrichment Score | Expression level  (RPKM value) |
| --- | --- | --- | --- |
| ATF2 | HepG2, K562 | 0.65, 0.43 | 35.35, 49.27 |
| ATF4 | K562 | 0.46 | 374.61 |
| ATF7 | HepG2 | 0.59 | 11.26 |
| CEBPB | K562, HepG2, H1, HeLa-S3 | 0.47, 0.26, 0.19,0.25 | 10.53,42.83, 13.88,29.7 |
| CEBPG | K562 | 0.52 | 33.14 |
| CTCF | HeLa-S3 | 0.13 | 41.4 |
| ESRRB | K562 | 0.14 | 11.63 |
| FOS | HeLa-S3 | 0.15 | 11.02 |
| FOXA2 | HepG2 | 0.15 | 10.88 |
| FOXA3 | K562 | 0.20 | 12.74 |
| GATA1 | K562 | 0.13 | 125.16 |
| HLF | HepG2 | 0.31 | 180.96 |
| HMBOX1 | K562 | 0.24 | 27.41 |
| IRF3 | HeLa-S3 | 0.22 | 23.49 |
| JUN | HepG2 | 0.18 | 28.16 |
| JUND | HepG2, HeLa-S3 | 0.17, 0.15 | 46.02,14.06 |
| LEF1 | K562 | 0.13 | 13.49 |
| MAFF | HepG2 | 0.49 | 13.95 |
| MAFG | K562, HepG2 | 0.36, 0.14 | 26.12, 21.66 |
| NFATC3 | K562 | 0.14 | 56.33 |
| NFE2 | K562 | 0.18 | 143.18 |
| NFE2L1 | K562 | 0.13 | 28.47 |
| NFE2L2 | HepG2, HeLa-S3 | 0.21 ,0.13 | 75.31,87.55 |
| NFYB | HeLa-S3 | 0.14 | 21.26 |
| PBX2 | K562 | 0.27 | 49.68 |
| RBPJ | K562 | 0.16 | 22.70 |
| REST | H1, HeLa-S3 | 0.14,0.26 | 17.98,18.56 |
| RFX5 | HeLa-S3 | 0.18 | 57.07 |
| SPI1 | K562 | 0.14 | 12.52 |
| SREBF2 | HeLa-S3 | 0.15 | 23.51 |
| SRF | H1, K562, HepG2 | 0.30, 0.20, 0.14 | 28.78, 28.71, 51.26 |
| STAT3 | HeLa-S3 | 0.14 | 25.54 |
| USF1 | H1, HepG2 | 0.18, 0.15 | 15.91, 16.61 |
| USF2 | HeLa-S3 | 0.19 | 34.93 |
| ZKSCAN1 | HepG2, K562, HeLa-S3 | 0.25, 0.25,0.36 | 49.98, 29.79,14.09 |
| ZNF24 | K562, HepG2 | 0.26, 0.15 | 53.34, 19.30 |
| ZNF274 | K562, HepG2, HeLa-S3 | 1.35, 0.14,5.4 | 27.04, 15.37,13.84 |
| ZNF282 | HepG2 | 0.16 | 11.46 |
| ZNF460 | HepG2 | 0.13 | 12.67 |

**Supplementary file 1—table 3.** Transcription factors that have binding sites enriched on nucleosomal regions located in the differentially open chromatin regions (Enrichment score >=1 and q-value <=0.05).

| TF name | Cell line | Enrichment score | TF name | Cell line | Enrichment Score |
| --- | --- | --- | --- | --- | --- |
| ATF4 | K562 | 1.44 | **LEF1** | K562 | 4.48 |
| BACH1 | K562 | 2.20 | **MAFF** | HeLa-S3,HepG2,K562 | 1.38,1.25,2.35 |
| BCL6 | HepG2 | 1.17 | **MAFG** | HepG2,K562 | 1.28,2.79 |
| CEBPA | HepG2 | 2.95 | **MAFK** | K562 | 1.90 |
| CEBPB | HeLa-S3,HepG2,K562 | 2.58,1.68,1.26 | **MEF2A** | HepG2,K562 | 1.95,2.51 |
| CEBPD | HepG2 | 2.84 | **MEF2D** | K562 | 3.11 |
| CEBPG | HepG2,K562,MCF-7 | 1.92,1.29,1.35 | **MEIS2** | K562 | 1.11 |
| CUX1 | K562 | 1.29 | **NFATC3** | K562 | 1.76 |
| ESRRA | HepG2 | 1.42 | **NFE2** | K562 | 2.28 |
| FOS | HeLa-S3,K562,MCF-7 | 1.25,1.91,1.78 | **NFE2L1** | K562 | 2.75 |
| FOSL1 | K562 | 1.89 | **NFIC** | HepG2 | 1.86 |
| FOSL2 | HepG2,MCF-7 | 1.46,1.76 | **NFIL3** | HepG2 | 1.80 |
| FOXA1 | HepG2,K562,MCF-7 | 2.76,4.27,2.78 | **NFIX** | K562 | 1.46 |
| FOXA2 | HepG2 | 2.26 | **NR2F1** | HepG2,K562 | 1.61,1.08 |
| FOXA3 | HepG2,K562 | 2.01,2.61 | **NR2F2** | HepG2,K562 | 2.70,1.75 |
| FOXK2 | K562 | 1.35 | **NR4A1** | K562 | 1.87 |
| FOXP1 | HepG2 | 2.38 | **NR5A1** | HepG2 | 1.81 |
| GATA1 | K562 | 6.15 | **ONECUT1** | HepG2 | 1.72 |
| GATA2 | HepG2,K562 | 4.51,6.39 | **ONECUT2** | HepG2 | 2.33 |
| GATA3 | MCF-7 | 2.14 | **PBX2** | HepG2,K562 | 1.70,1.87 |
| GATA4 | HepG2 | 2.54 | **PPARG** | HepG2 | 1.13 |
| GFI1 | HepG2 | 1.14 | **PRDM1** | HeLa-S3 | 1.60 |
| HLF | HepG2 | 1.41 | **RARA** | HepG2 | 1.95 |
| HMBOX1 | K562 | 1.55 | **RUNX1** | K562 | 2.93 |
| HNF1A | HepG2 | 4.09 | **RXRB** | HepG2 | 2.03 |
| HNF1B | HepG2 | 1.55 | **SOX13** | HepG2 | 2.43 |
| HNF4A | HepG2 | 3.64 | **SRF** | K562 | 2.05 |
| HNF4G | HepG2 | 3.32 | **STAT3** | HeLa-S3 | 4.25 |
| IKZF1 | K562 | 1.35 | **TCF7** | HepG2,K562 | 3.51,1.30 |
| IRF1 | K562 | 1.31 | **TCF7L2** | HepG2,MCF-7 | 2.73,1.42 |
| IRF9 | K562 | 2.57 | **TEAD1** | HepG2 | 1.36 |
| JUN | HeLa-S3 | 1.22 | **TEF** | HepG2 | 1.61 |
| JUNB | K562 | 2.06 | **THRB** | HepG2 | 2.15 |
| JUND | HeLa-S3,HepG2,K562 | 2.27,1.44,1.97 | **ZNF24** | K562 | 1.13 |
| LEF1 | K562 | 4.48 | **ZNF652** | HepG2 | 1.30 |
| KLF10 | K562 | 3.31 |  |  |  |

**Supplementary file 1—table 4.** Predicted potential pioneer factors from enrichment analysis using the NRs located in differentially open chromatin regions and NDRs located in conserved open chromatin regions. Transcription factors that have binding sites enriched on nucleosomal regions in the differentially open chromatin regions (Enrichment score >=1 and q-value <=0.05) and are significantly expressed (RPKM >=10) in corresponding cell lines.

| TF name | Cell line | Enrichment score | Expression level (RPKM value) |
| --- | --- | --- | --- |
| ATF4 | K562 | 1.44 | 374.61 |
| BACH1 | K562 | 2.20 | 21.38 |
| BCL6 | HepG2 | 1.17 | 28.63 |
| CEBPA | HepG2 | 2.95 | 33.37 |
| CEBPB | HeLa-S3,HepG2,K562 | 2.58,1,68,1.26 | 29.70,42.83,10.53 |
| CEBPD | HepG2 | 2.84 | 26.11 |
| CEBPG | HepG2,K562 | 1.92,1.29 | 31.21,33.14 |
| ESRRA | HepG2 | 1.42 | 16.10 |
| FOS | HeLa-S3 | 1.25 | 11.02 |
| FOSL2 | HepG2 | 1.46 | 19.26 |
| FOXA1 | HepG2 | 2.76 | 32.38 |
| FOXA2 | HepG2 | 2.26 | 10.88 |
| FOXA3 | HepG2,K562 | 2.01,2.61 | 57.20, 12.74 |
| FOXK2 | K562 | 1.35 | 22.64 |
| GATA1 | K562 | 6.15 | 125.16 |
| GATA2 | K562 | 6.39 | 18.36 |
| GATA4 | HepG2 | 2.54 | 13.42 |
| HLF | HepG2 | 1.41 | 180.96 |
| HMBOX1 | K562 | 1.55 | 27.41 |
| HNF4A | HepG2 | 3.64 | 72.69 |
| IKZF1 | K562 | 1.35 | 14.53 |
| JUNB | K562 | 2.06 | 19.14 |
| JUND | HeLa-S3,HepG2,K562 | 2.27,1.44,1.97 | 14.06,46.02,41.06 |
| LEF1 | K562 | 4.48 | 13.49 |
| MAFF | HepG2 | 1.25 | 13.95 |
| MAFG | HepG2,K562 | 1.28,2.79 | 21.66,26.12 |
| MEF2A | HepG2 | 1.95 | 14.56 |
| NFATC3 | K562 | 1.76 | 56.33 |
| NFE2 | K562 | 2.28 | 143.18 |
| NFE2L1 | K562 | 2.75 | 28.47 |
| NFIL3 | HepG2 | 1.80 | 51.02 |
| NR2F1 | HepG2 | 1.61 | 28.15 |
| NR5A1 | HepG2 | 1.81 | 28.13 |
| PBX2 | HepG2,K562 | 1.70,1.87 | 37.77,49.68 |
| PPARG | HepG2 | 1.13 | 26.05 |
| RXRB | HepG2 | 2.03 | 34.15 |
| SRF | K562 | 2.05 | 28.71 |
| STAT3 | HeLa-S3 | 4.25 | 25.54 |
| TEAD1 | HepG2 | 1.36 | 13.31 |
| ZNF24 | K562 | 1.13 | 53.34 |

**Supplementary file 1—table 5.** A list of well-characterized known pioneer transcription factors (PTFs) from multiple literature studies.

| Transcription Factor Name | Function |
| --- | --- |
| FOXA1, FOXA2, FOXA3 | Tissue-specific gene activation; embryonic development, establishment of tissue-specific gene expression and regulation of gene expression in differentiated tissues. |
| GATA1, GATA2, GATA3, GATA4 | Early stages of cell differentiation and organ development across a variety of tissues; development and maintenance of hematopoietic systems. |
| CEPBA, CEBPB | Fate decisions during myeloid differentiation; essential for maintaining homeostasis of both embryonic and adult tissues; key regulators of hepatocyte differentiation. |
| NFY complex: NFYA, NFYB, NFYC | NF-Y, also known as the CCAAT-binding factor CBF, is a ubiquitously expressed heterotrimeric TF composed of NF-YA, NF-YB, and NF-YC subunits. Cell type-specific master transcription factors; NF-Y complex is required for the maintenance of embryonic stem cell (ESC) identity and is an essential component of the core pluripotency network. |
| ESRRB | Suppress cell differentiation and sustain ESC self-renewal |
| POU5F1 (OCT-4) | Yamanaka transcription factors  cellular reprogramming of somatic cells into induced pluripotent stem cells. |
| KLF4 |  |
| NEUROD1 | Neuronal reprogramming/neuronal conversion from human fibroblasts; neuronal differentiation. |
| TP53 | DNA repair, cell-cycle arrest, and apoptosis; tumor suppressor. |
| AP-1 complex: FOS, FOSL, FOSL2, MAFG, MAFF, MAFK, JUN, JUNB, JUND, ATF2, ATF3, ATF4, ATF6, ATF7 | The transcription factor AP-1 is a heterodimeric protein, composed of members of the basic region leucine zipper protein superfamily, specifically, the JUN, FOS, and activating transcription factor proteins, which regulate gene expression in response to a variety of stimuli, including cytokines, growth factors, stress, and bacterial and viral infections. |
| SPI1(PU.1) | PU.1 is an ETS-family transcription factor that plays a broad range of roles in hematopoiesis. A direct regulator of myeloid, dendritic-cell, and B cell functional programs, and a well-known antagonist of terminal erythroid cell differentiation. |

**Supplementary file 1—table 6.** Three groups of known pioneer factors for validation of enrichment analysis.

| Test set 1: 32 known pioneer factors | FOXA1, FOXA2, FOXA3, GATA1, GATA2, GATA3, GATA4, CEPBA, CEBPB, NFYA, NFYB, NFYC, ESRRB, POU5F1, KLF4, NEUROD1, TP53, FOS, FOSL1, FOSL2, MAFG, MAFF, MAFK, JUN, JUNB, JUND, ATF2, ATF3, ATF4, ATF6, ATF7, SPI1 |
| --- | --- |
| Test set 2: 11 known pioneer factors with essential roles in cell differentiation | FOXA1, FOXA2, FOXA3, GATA1, GATA2, GATA3, GATA4, CEPBA, CEBPB, NEUROD1, SPI1 |
| Test set 3: 7 known pioneer factors for the maintenance of embryonic stem cell or reprogramming of somatic cells into induced pluripotent stem cells | NFYA, NFYB, NFYC, ESRRB, NEUROD1, KLF4, POU5F1 |

**Supplementary file 1—table 7.** Performance of enrichment scores in the classification of pioneer factors.

| NR and NDR | Validation Set | Significantly Expressed TFs | ROC-AUC | pr-ROC-AUC | MCC (maximum value) | Percentage of TFs as True Positives |
| --- | --- | --- | --- | --- | --- | --- |
| All | Test Set 1 | No | 0.69 | 0.33 | 0.31 | 0.17 |
|  | Test Set 1 | Yes | **0.71** | **0.37** | **0.31** | 0.2 |
|  | Test Set 2 | No | 0.77 | 0.1 | 0.22 | 0.04 |
|  | Test Set 2 | Yes | 0.76 | 0.13 | 0.22 | 0.06 |
| Differentially and conserved open chromatin regions | Test Set 1 | No | 0.71 | 0.34 | 0.28 | 0.16 |
|  | Test Set 1 | Yes | 0.71 | 0.41 | 0.33 | 0.2 |
|  | Test Set 2 | No | 0.89 | 0.41 | 0.42 | 0.04 |
|  | Test Set 2 | Yes | **0.92** | **0.45** | **0.49** | 0.06 |

**Supplementary file 1—table 8.** List of TFs that have been previously suggested *as potential pioneer factors* and/or *nucleosome binders* from the literature.

| TF name | Function | Reference |
| --- | --- | --- |
| LEF1 | able to bind with HIV-1 nucleosome core particles | doi:10.1093/emboj/16.9.2463 |
| HNF4A | a potential pioneer factor in remodeling the active chromatin landscape in the liver | doi:10.1038/s41467-021-26567-3 |
| PBX2 | one PBC transcription factor which could have pioneer activity | doi:10.3389/fcell.2017.00009 |
| NR5A1 | considered as a pioneer transcription factor for Leydig cell development and function | doi:10.1016/j.stemcr.2020.07.002 |
| NFE2 | interact with the cognate motif on the nucleosome before chromatin is remodeled | doi:10.1074/jbc.M209612200 |
| CUX1 | specifically interact with its recognition motif in a nucleosomal context without displacing the nucleosome core | doi:10.1023/a:1007058123699 |
| FOXK2 | possibly act as an early pioneer factor in gene activation during cell differentiation | doi:10.1093/nar/gkab221 |
| NRF1 | predicted on theoretical bases to have pioneer action and trigger chromatin access (DNase sensitivity) only if its DNA-binding site is unmethylated | doi:10.1074/jbc.R117.001232 |
| RFX5 | displace nucleosomes | doi:10.1093/nar/gkad614 |
| CREB1 | bind to inaccessible chromatin regions | doi:10.1093/nar/gkad614 |
| FOXO4 | potential pioneer factors in cancer development | doi:10.1074/jbc.R117.001232 |
| CLOCK | CLOCK:BMAL1 DNA binding promotes rhythmic chromatin opening | doi:10.1101/gad.228536.113 |

**Supplementary file 1—table 9.** Predicted potential pioneer factors from the association analysis between the binding motif profiles and nucleosome occupancy values. A list of TFs with statistically significant positive correlations between the TF binding motif profiles and nucleosome occupancy levels (p-value <=0.05). TF expression level data is not available for MCF-7 cell line.

| TF name | Cell Line | PCC | Expression level |
| --- | --- | --- | --- |
| ZKSCAN1 | HeLaS3, K562, MCF7, HepG2 | 0.75,0.64,0.63, 0.34 | 14.1,29.8, NA, 50.0 |
| ESR1 | MCF7 | 0.58 | NA |
| NFATC3 | K562 | 0.56 | 56.3 |
| ZBTB7B | MCF7, HepG2 | 0.55,0.37 | NA, 15.0 |
| MAX | H1 | 0.51 | 12.4 |
| TBX2 | HepG2 | 0.50 | 2.2 |
| NFIB | MCF7 | 0.48 | NA |
| USF1 | H1 | 0.46 | 15.9 |
| CUX1 | MCF7, K562 | 0.41,0.08 | NA, 2.4 |
| ZNF274 | HepG2 | 0.37 | 15.4 |
| SREBF1 | MCF7 | 0.36 | NA |
| NFYB | HeLaS3 | 0.33 | 21.3 |
| ZNF24 | MCF7, K562 | 0.32,0.08 | NA, 53.3 |
| NR2F2 | MCF7 | 0.31 | NA |
| ZNF282 | K562 | 0.30 | 17.7 |
| RELA | K562 | 0.30 | 8.9 |
| USF2 | HeLaS3 | 0.28 | 34.9 |
| ESRRA | MCF7 | 0.28 | NA |
| POU5F1 | H1 | 0.26 | 183.0 |
| NFKB2 | HepG2 | 0.22 | 11.1 |
| BACH1 | H1 | 0.22 | 6.3 |
| SREBF2 | HeLaS3 | 0.21 | 23.5 |
| TEAD4 | H1 | 0.20 | 44.0 |
| MIXL1 | HepG2 | 0.18 | 9.76 |
| NR4A1 | K562 | 0.15 | 0.8 |
| E2F6 | H1 | 0.15 | 3.1 |
| RARA | HepG2 | 0.14 | 2.7 |
| CEBPB | HeLaS3 | 0.13 | 29.7 |
| SMAD5 | K562 | 0.12 | NA |
| NFYA | HeLaS3 | 0.11 | 12.5 |
| HMBOX1 | K562 | 0.11 | 27.4 |
| ZNF460 | HepG2 | 0.11 | 12.7 |
| CTCF | H1 | 0.10 | 30.7 |
| EGR1 | H1 | 0.10 | 4.80 |
| GATA3 | MCF7 | 0.09 | NA |
| ATF2 | K562 | 0.08 | 49.3 |
| TCF7L2 | MCF7 | 0.07 | NA |

**Supplementary file 1—table 10.** Summary of MNase-seq experimental data for genome-wide nucleosome mapping.

| Cell lines | Sequencing type | GEO Accession number |
| --- | --- | --- |
| MCF-7 breast cancer cell line | paired-end | GSE51097 |
| K562 immortalized myelogenous leukemia cell line | paired-end | GSE78984 |
| H1 human embryonic stem cell line | paired-end | GSM1194220 |
| HepG2 human liver cancer cell line | paired-end | GSM3718063 |
| HeLa immortalized cervical tumor cell line | paired-end | GSE100401 |

**Supplementary file 1—table 11.** Summary of the number of identified nucleosome regions (NRs) in each cell line using the reads with fragment sizes between 146-148.

| Cell lines | number of NRs |
| --- | --- |
| MCF-7 breast cancer cell line | 10,290,828 |
| K562 immortalized myelogenous leukemia cell line | 2,780,423 |
| H1 human embryonic stem cell line | 12,551,429 |
| HepG2 human liver cancer cell line | 5,193,194 |
| HeLa immortalized cervical tumor cell line | 9,842,040 |

**Supplementary file 1—table 12.** List of the analyzed transcription factors and corresponding identifiers of position frequency matrices (PFMs) files from the JASPER database.

| TF gene name | Motif ID | TF gene name | Motif ID | TF gene name | Motif ID | TF gene name | Motif ID |
| --- | --- | --- | --- | --- | --- | --- | --- |
| ATF2 | MA1632.1 | **GABPA** | MA0062.3 | **MYBL2** | MA0777.1 | **SPDEF** | MA0686.1 |
| ATF3 | MA0605.2 | **GATA1** | MA0035.4 | **MYC** | MA0147.3 | **SPI1** | MA0080.5 |
| ATF4 | MA0833.2 | **GATA2** | MA0036.3 | **NEUROD1** | MA1109.1 | **SREBF1** | MA0595.1 |
| ATF6 | MA1466.1 | **GATA3** | MA0037.3 | **NFATC3** | MA0625.1 | **SREBF2** | MA0596.1 |
| ATF7 | MA0834.1 | **GATA4** | MA0482.2 | **NFE2** | MA0841.1 | **SRF** | MA0083.3 |
| BACH1 | MA1633.1 | **GFI1** | MA0038.2 | **NFE2L1** | MA0089.2 | **STAT3** | MA0144.2 |
| BCL6 | MA0463.2 | **GMEB2** | MA0862.1 | **NFE2L2** | MA0150.1 | **TBX18** | MA1565.1 |
| BHLHE40 | MA0464.2 | **HES1** | MA1099.2 | **NFIA** | MA0670.1 | **TBX2** | MA0688.1 |
| CEBPA | MA0102.4 | **HEY1** | MA0823.1 | **NFIB** | MA1643.1 | **TBX3** | MA1566.1 |
| CEBPB | MA0466.2 | **HINFP** | MA0131.2 | **NFIC** | MA0161.2 | **TCF3** | MA0522.3 |
| CEBPD | MA0836.2 | **HLF** | MA0043.3 | **NFIL3** | MA0025.2 | **TCF7** | MA0769.2 |
| CEBPG | MA0838.1 | **HMBOX1** | MA0895.1 | **NFIX** | MA0671.1 | **TCF7L2** | MA0523.1 |
| CLOCK | MA0819.1 | **HNF1A** | MA0046.2 | **NFKB2** | MA0778.1 | **TCFL5** | MA0632.2 |
| CREB1 | MA0018.4 | **HNF1B** | MA0153.2 | **NFYA** | MA0060.3 | **TEAD1** | MA0090.3 |
| CREB3 | MA0638.1 | **HNF4A** | MA0114.4 | **NFYB** | MA0502.2 | **TEAD2** | MA1121.1 |
| CREB3L1 | MA0839.1 | **HNF4G** | MA0484.2 | **NFYC** | MA1644.1 | **TEAD4** | MA0809.2 |
| CREM | MA0609.2 | **HOXA5** | MA0158.2 | **NKX3-1** | MA0124.1 | **TEF** | MA0843.1 |
| CTCF | MA0139.1 | **HSF1** | MA0486.2 | **NR2C1** | MA1535.1 | **TFAP4** | MA0691.1 |
| CTCFL | MA1102.2 | **HSF2** | MA0770.1 | **NR2C2** | MA0504.1 | **TFCP2** | MA0145.3 |
| CUX1 | MA0754.1 | **IKZF1** | MA1508.1 | **NR2F1** | MA0017.2 | **TFDP1** | MA1122.1 |
| DBP | MA0639.1 | **IRF1** | MA0050.2 | **NR2F2** | MA1111.1 | **TFE3** | MA0831.2 |
| DLX6 | MA0882.1 | **IRF2** | MA0051.1 | **NR3C1** | MA0113.3 | **TF_name** | Motif_ID |
| E2F1 | MA0024.3 | **IRF3** | MA1418.1 | **NR4A1** | MA1112.2 | **TGIF2** | MA0797.1 |
| E2F2 | MA0864.2 | **IRF5** | MA1420.1 | **NR5A1** | MA1540.1 | **THAP1** | MA0597.1 |
| E2F3 | MA0469.3 | **IRF9** | MA0653.1 | **NRF1** | MA0506.1 | **THAP11** | MA1573.1 |
| E2F4 | MA0470.2 | **ISL2** | MA0914.1 | **NRL** | MA0842.2 | **THRB** | MA1574.1 |
| E2F6 | MA0471.2 | **ISX** | MA0654.1 | **ONECUT1** | MA0679.2 | **TP53** | MA0106.3 |
| E2F7 | MA0758.1 | **JUN** | MA0488.1 | **ONECUT2** | MA0756.1 | **USF1** | MA0093.3 |
| E2F8 | MA0865.1 | **JUNB** | MA0490.2 | **OVOL1** | MA1544.1 | **USF2** | MA0526.3 |
| EGR1 | MA0162.4 | **JUND** | MA0491.2 | **PBX2** | MA1113.2 | **VEZF1** | MA1578.1 |
| ELF1 | MA0473.3 | **KLF10** | MA1511.1 | **PITX1** | MA0682.2 | **XBP1** | MA0844.1 |
| ELF2 | MA1483.1 | **KLF11** | MA1512.1 | **PKNOX1** | MA0782.2 | **YY1** | MA0095.2 |
| ELF3 | MA0640.2 | **KLF13** | MA0657.1 | **POU5F1** | MA1115.1 | **ZBED1** | MA0749.1 |
| ELF4 | MA0641.1 | **KLF16** | MA0741.1 | **PPARG** | MA0066.1 | **ZBTB12** | MA1649.1 |
| ELK1 | MA0028.2 | **KLF4** | MA0039.4 | **PRDM1** | MA0508.3 | **ZBTB14** | MA1650.1 |
| ELK3 | MA0759.1 | **KLF6** | MA1517.1 | **PRDM4** | MA1647.1 | **ZBTB26** | MA1579.1 |
| ELK4 | MA0076.2 | **KLF9** | MA1107.2 | **RARA** | MA0729.1 | **ZBTB33** | MA0527.1 |
| ERF | MA0760.1 | **LBX2** | MA0699.1 | **RBPJ** | MA1116.1 | **ZBTB7A** | MA0750.2 |
| ESR1 | MA0112.3 | **LEF1** | MA0768.1 | **RELA** | MA0107.1 | **ZBTB7B** | MA0694.1 |
| ESRRA | MA0592.3 | **MAFF** | MA0495.3 | **REST** | MA0138.2 | **ZEB1** | MA0103.3 |
| ESRRB | MA0141.3 | **MAFG** | MA0659.2 | **RFX1** | MA0509.2 | **ZKSCAN1** | MA1585.1 |
| ETS1 | MA0098.3 | **MAFK** | MA0496.3 | **RFX3** | MA0798.2 | **ZKSCAN5** | MA1652.1 |
| ETS2 | MA1484.1 | **MAX** | MA0058.3 | **RFX5** | MA0510.2 | **ZNF143** | MA0088.2 |
| ETV1 | MA0761.2 | **MAZ** | MA1522.1 | **RFX7** | MA1554.1 | **ZNF148** | MA1653.1 |
| ETV4 | MA0764.2 | **MEF2A** | MA0052.4 | **RORA** | MA0071.1 | **ZNF24** | MA1124.1 |
| ETV5 | MA0765.2 | **MEF2D** | MA0773.1 | **RREB1** | MA0073.1 | **ZNF263** | MA0528.2 |
| ETV6 | MA0645.1 | **MEIS1** | MA0498.2 | **RUNX1** | MA0002.1 | **ZNF274** | MA1592.1 |
| FOS | MA0476.1 | **MEIS2** | MA0774.1 | **RXRB** | MA0855.1 | **ZNF282** | MA1154.1 |
| FOSL1 | MA0477.2 | **MGA** | MA0801.1 | **SIX1** | MA1118.1 | **ZNF317** | MA1593.1 |
| FOSL2 | MA0478.1 | **MITF** | MA0620.3 | **SMAD3** | MA0795.1 | **ZNF382** | MA1594.1 |
| FOXA1 | MA0148.4 | **MIXL1** | MA0662.1 | **SMAD5** | MA1557.1 | **ZNF384** | MA1125.1 |
| FOXA2 | MA0047.3 | **MLX** | MA0663.1 | **SNAI1** | MA1558.1 | **ZNF460** | MA1596.1 |
| FOXA3 | MA1683.1 | **MNT** | MA0825.1 | **SOX13** | MA1120.1 | **ZNF652** | MA1657.1 |
| FOXK1 | MA0852.2 | **MNX1** | MA0707.1 | **SOX18** | MA1563.1 | **ZNF740** | MA0753.2 |
| FOXK2 | MA1103.2 | **MSX2** | MA0708.1 | **SP1** | MA0079.4 | **ZSCAN29** | MA1602.1 |
| FOXO4 | MA0848.1 | **MTF1** | MA0863.1 | **SP2** | MA0516.2 |  |  |
| FOXP1 | MA0481.3 | **MXI1** | MA1108.2 | **SP4** | MA0685.1 |  |  |

**Supplementary file 1—table 13.** A contingency table for binding enrichment analysis to quantify the TF’s binding preferences on NRs or NDRs. Numbers are given in base pairs.

|  | **ChIP-seq TF motifs** | **Outside of ChIP-seq TF motifs** |
| --- | --- | --- |
| **Nucleosome Regions** | a | b |
| **Nucleosome Depleted Regions** | c | d |
